# Relationship between periodontal variables and disease severity in patients with chronic obstructive pulmonary disease

**DOI:** 10.15171/japid.2018.001

**Published:** 2018-06-20

**Authors:** Amir Moeintaghavi, Shahrzad Mohammadzadeh Lari, Farid Shiezadeh, Zakieh Mohammadian, Shamim Tajik, Nahid Nasrabadi

**Affiliations:** ^1^Dental Research Center, Mashhad University of Medical Sciences, Mashhad, Iran; ^2^Department of Pulmonology, Mashhad University of Medical Sciences, Mashhad, Iran; ^3^Department of Periodontics, Mashhad University of Medical Sciences, Mashhad, Iran; ^4^Private Practice, Mashhad, Iran; ^5^Mashhad University of Medical Sciences, Mashhad, Iran

**Keywords:** Periodontal pocket depth, periodontal parameters, COPD, FEV1, periodontal disease

## Abstract

**Background:**

The present study investigated the relationship between certain periodontal variables and severity of disease in COPD patients.

**Methods:**

The present cross-sectional study included 50 patients suffering from COPD. Lung function examination, forced expiratory volume in one second (FEV1), forced vital capacity (FVC), FEV1/FVC, SpO2, and Modified Medical Research Council (MMRC) Dyspnea Scale were performed. Periodontal clinical examination index included probing depth (PD), attachment loss (AL), gingival index (GI) and plaque index (PI). A quality of life validated index, the COPD assessment test (CAT index), was also calculated.

**Results:**

The FEV1 and FVC indices showed a significant negative correlation with PI and AL variables only. The COPD assessment test (CAT) index showed a significant but positive correlation with PI and AL variables only. The SpO2 index presented a significant negative correlation with GI and AL variables. The FEV1/FVC ratio was found to have a negative correlation with PD and AL variables. It is worth noting that MMRC exhibited no significant relationship with any of the periodontal variables. The only variable that was significantly different (P=0.022) among the three smoking groups was the FVC index. The FVC value was significantly higher in the group of subjects who smoked more than 10 cigarettes per day versus the non-smoking group (P=0.017).

**Conclusion:**

Based on the findings of this study, in view of the relationship between periodontal variables and respiratory indices in the course of COPD, early treatment of periodontal diseases, might considerably reduce the severity of COPD.

## Introduction

In recent years an enormous body of research has emphasized the significant role that oral hygiene plays in the development of pulmonary diseases, including COPD. Some studies have even identified the oral cavity as the main source and origin of pulmonary pathogens and have presented certain mechanisms to explain this phenomenon.^1^ Many studies have focused on the association between COPD and periodontal diseases. However, there is scant data available on the correlation between the severity of COPD and periodontal variables.

Periodontal diseases are inflammatory disorders that involve the supporting tissues of the tooth, and are generally caused by specific microorganisms or a group of microorganisms. Periodontitis is recognized as the chronic inflammatory response of the periodontium to bacterial infection, which can lead to the gradual loss of the supporting structures along with alveolar ridge resorption, eventually resulting in tooth mobility and tooth loss.^2, 3^ The occurrence of periodontitis means that specialized indices, such as gingival index (GI), plaque index (PI), probing depth (PD) and attachment loss (AL)are out of their normal range.

Chronic obstructive pulmonary disease (COPD) is a term that refers to a number of progressive lung disorders characterized by reduced maximum expiratory pressure (MEP). COPD includes chronic bronchitis and emphysema. Although these two are distinct processes, they typically coincide. In pathological terms, emphysema occurs when the air spaces beyond the terminal bronchiole are permanently dilated and damaged, accompanied by the destruction of alveolar walls and without obvious fibrosis.^4^ COPD is associated with various risk factors, among which smoking is the most frequent.^5, 6^

Smokers account for approximately 90% of COPD patients. Smoking is not only the most common individual factor that leads to chronic obstruction of air passages, but is also responsible for elevated impact of other identified risk factors, such as air pollution, hazardous workplace and infection.^7^

Various studies have examined infected lungs by performing biopsy. They compared the data collected from samples of the oral flora. The results revealed similarities between the microorganisms found in the two sets of samples. Accordingly, it has been suggested that the oral cavity might serve as a potential source of pathogens that cause pulmonary infections and COPD.^8^

Aspiration of oropharyngeal contents is widely recognized as the most frequent cause of pulmonary infection.^1, 9^

A study conducted by Scannapieco et al (1998) showed that people with poor oral hygiene are more likely to suffer from respiratory disorders. The results demonstrated thatthose with high OHI scores were 4.3 times more likely to develop chronic respiratory disorders.^10^

Xuan Zhou et al^11^ reported that poor periodontal health had a significant correlation with low quality of life in COPD patients.

Shen et al^12^ evaluated the relationship between COPD and periodontitis via a retrospective cohort study which enrolled a relatively large population. They recruited 5562 COPD patients with periodontal diseases, who had received periodontal treatment; they formed the treatment group. The controls were selected at a 1:1 ratio and were matched by the propensity score estimated with age, sex, dates of COPD diagnosis and periodontal treatment, as well as co-morbidities. Both groups were followed up for 5 years. Eventually, 0.57 adjusted hazard ratio was reported (95% confidence interval: 0.52‒0.62) andit was reported that periodontal treatment of COPD patients could reduce the risk of adverse respiratory events and mortality.

As a result, the relationship between COPD and periodontal disease has been examined in numerous studies, but to the best of our knowledge periodontal parameters and severity of disease in COPD patients has not been evaluated.

The present study investigated the relationship between certain periodontal variables (GI, PI, ALand PD) and severity of disease in COPD patients.

## Methods

The present cross-sectional study included 50 patients suffering from COPD, who had been referred to the SpecialtyClinic of Gha'emHospital from September 2016 to June 2017. These participants, who had been diagnosed with COPD by a pulmonologist (based on American Thoracic Society (ATS) guideline) in the SpecialtyClinic of Gha'emHospital, were included in the study after being informed about the study procedures and signing written consent forms. Data werecollected using questionnaires, interviews, clinical examinations and preclinical tests. In addition, the information regarding the participants’ age, gender, location of residence and smoking status was obtained through oral questions.

Patients who were edentulous ordiabetic, those who took medications that affectedthe periodontium (such as cyclosporine, nifedipine andphenytoin) and pregnant womenwere excluded from the study.

Data concerning pulmonary function tests were registered using a spirometer. These tests included:

### Forced expiratory volume in one second (FEV1)

It measures how much air a person can exhale during the first second of a forced breath following a maximum inhalation. FEV1 is reversely correlated with the severity of COPD. Based on this index, COPD patients can be classified into several grades as follows:

1. Mild (stage I: FEV1≥80%)
2. Moderate (stage II: 50%≤FEV1<80%)
3. Severe (stage III: 30%≤FEV1<50%)
4. Very Severe (stage IV: FEV1<30%)

In all cases, FEV1/FVC <0.70 is considered the norm.

### Forced vital capacity (FVC)

It is a measure of your vital lung capacity and represents the volume of air which can be forcibly exhaled from the lungs after taking the deepest breath possible.

### FEV1/FVC ratio

It represents the percentage of the vital capacity that can be forcibly exhaled in one second. A healthy young adult should correspond to an approximate ratio of 85%, which gradually declines with age. The lowest normal range is considered to be 70‒75%.

### SpO_2_

SpO_2_ stands for peripheral capillary oxygen saturation – an estimate of the amount of oxygen in the blood, which was measured using a pulse oximeter.

### Modified medical research council (MMRC) dyspnea scale

It measures a person's degree of breathlessness on a scale of 0‒4 and is closely proportional to other health status measures and morbidity baseline. It uses the final value to determine how much disability is caused by shortness of breath (Table 1).

**Table 1:** mMRC test index representing the patient’s shortness of breath on a scale of 0 to 4

**mMRC grade 0**	only breathless with strenuous exercise
**mMRC grade 1**	gets short of breath when hurrying on the level or waking up slight hill
**mMRC grade 2**	walks slower than people of the same age on the level because of breathlessness, or has to stop for breath when waking on my pace on the level.
**mMRC grade 3**	stops for breath after walking about 100 meters or after a few minutes on the level.
**mMRC grade 4**	too breathless to leave the house or breathless when dressing or undressing.

### Quality of life (QoL)

It reflects the general well-being of individuals inhabiting a particular country or region. Generally, QoL can be quantified using subjective and objective indices. The scores attributed to this measure, which approximately describe the patients’ disease status, are provided by subjects via a test, commonly referred to as the COPD assessment test (CAT index). The scores vary on a scale of 0‒40.^11^

During the final stage, periodontal examination was carried out using a probe and mirror under a reading lamp. The following periodontal variables were evaluated:

### Probing depth (PD)

It represents the distance between the gingival margin and the deepest area of the pocket. Depth of the pocket was measured for every single tooth at four points (mesiobuccal, mid-buccal, distobuccal, and lingual/palatal) using Williams probe. The mean of the recorded numbers was considered as the probing depth and was registered for each patient.

Also, plaque index and gingival index (Sillness and Loe)^12^ were measured and the means of the recorded numbers were considered as the plaque index and gingival index and registered for each subject.

### Attachment loss (AL)

Attachment lossrepresents the distance between the CEJ and the deepest aspect of the pocket. Williams probe was employed to examine every tooth of each patient at four points to measure the distance between CEJ and the depth of the gingival sulcus. The mean of the recorded numbers was considered as the degree of periodontal attachment loss and registered for each participant.

### Statistical Analysis

Appropriate tables and diagrams were used to illustrate the study data. One-way ANOVA was used for data analysis.

### Ethical considerations

At the beginning of the project, the researcher fully introduced herself to the participants. The process and methodology of the experiment were explained to the patients in detail. This was followed by collecting written informed consent forms from the subjects. The current study was presented to the Ethics Committee of Mashhad University of Medical Sciencesin accordance with the World Medical Association Declaration of Helsinkiand was approved under codeIR.mums.sd.REC.1394.161.

## Results

The data relevant to qualitative and quantitative variables are summarized in Tables 2 and 3, respectively.

Based on the findings of this study, the FEV1 and FVC indicesexhibited a significant negative correlation with PI and AL variables only. Likewise, the COPD assessment test (CAT) showed a significant but positive correlation with PI and AL variables only. The SpO_2_ index had a significant negative correlation with GI and AL variables. The FEV1/FVC ratio was found to have a negative correlation with PD and AL variables. It is worth noting that MMRC exhibited no significant relationship with any of the periodontal variables (Table 4).

**Table 2:** Distribution of qualitative variables

**Variable**		**Frequency**	**Percentage**
**Gender**	**Female**	19	38
	**Male**	31	62
**Smoking status**	**None**	18	36
	**<10 cigarettes per day**	7	14
	**>10 cigarettes per day**	25	50
**Under medication**	**No**	22	44
	**Yes**	28	56

**Table 3:** Descriptive statistics of the variables (n=50)

**Variable**	**Mean**	**Standard Deviation**	**Minimum**	**Maximum**
**PD (mm)**	2.03	0.41	1.05	3.29
**AL (mm)**	3.40	1.30	0.05	6.00
**GI**	1.55	0.39	0.10	2.32
**PI**	1.64	0.49	0.11	2.41
**FEV1 (%)**	52.72	17.40	19.0	87.8
**FVC (%)**	68.30	18.16	29.0	99.0
**FEV1/FVC (%)**	62.80	9.92	33.0	81.0
**MMRC**	1.52	0.89	0	3
**SpO**_2_ ** (%)**	92.42	3.92	82	98
**CAT**	15.28	7.28	3.0	35.0

**Table 4:** Correlation coefficient between periodontal variables and COPD variables (n=50)

**Variable**	**PD**	**AL**	**GI**	**PI**
**FEV1**	- 0.162	- 0.481^*^ P<0.001	- 0.207	- 0.296^*^ P=0.037
**FVC**	- 0.107	- 0.370^*^ P=0.008	- 0.271	- 0.370^*^ P=0.008
**FEV1/FVC**	- 0.291^*^ P=0.04	- 0.470^*^ P=0.001	- 0.112	- 0.259
**MMRC**	0.204	0.210	0.236	0.262
**SpO**_2_	- 0.199	- 0.319^*^ P=0.024	- 0.339^*^ P=0.016	- 0.240
**CAT**	0.273	0.332^*^ P=0.018	0.160	0.378^*^ P=0.007

*Significant at 0.05 level

The mean GI value was significantly higher in females compared to males (P=0.045). In addition, the mean CAT value proved to be significantly greater in females compared to males (P=0.046). Comparison between the group under medication and the no-medication group revealed no significant difference between the mean values of the variables.

**Figure 1:**
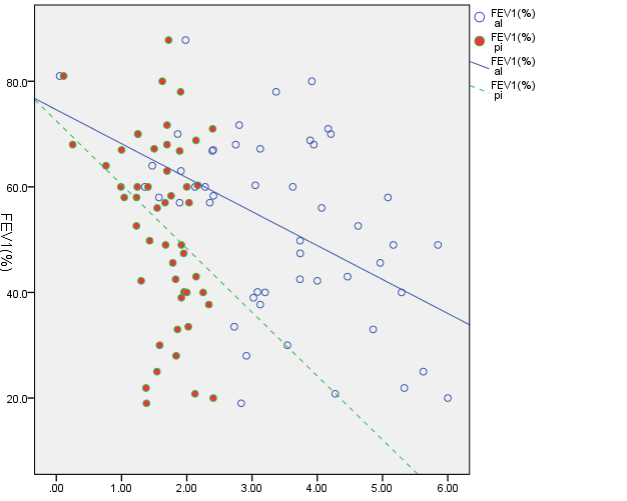
Scatter plot for FEV1 correlation with AL and PI variables.

The only variable that showed a significant difference (P=0.022) among the three smoking groups was the FVC index. Pair-wise comparisons of these groups revealed that the FVC value was significantly higher in the group with subjects who smoked >10 cigarettes a day compared to the non-smoking group (P=0.017) (Figure 1).

## Discussion

Chronic obstructive pulmonary disease (COPD) is a term used to describe a group of chronic, progressive lung diseases characterized by obstructed airflow from the lungs during exhalation, resulting in residual air. They are associated with abnormal inflammatory reactions of the lungs to airborne particles or inhaled fumes. COPD is believed to be among the leading causes of mortality and morbidity worldwide.^13^ The stable and exacerbated phases of COPD are both associated with inflammation. Pulmonary inflammation, in turn, accounts for a particular degree of systemic inflammation.^14^ In recent years, therefore, COPD has been regarded as a systemic disease, with certain inflammatory mediators being responsible for the systemic manifestations associated with COPD.^15, 16^

A meta-analysis conducted by Chen et al^19^ suggests that a high level of prostaglandin E2 (PGE2), as a proinflammatory mediator, is linked with the severity of airflow obstruction in stable COPD. Thus, it is plausible to point out that PGE2 is a significant contributing factor in the pathogenesis of inflammation in COPD. Among proinflammatory mediators, PGE2 and IL-1β play a major role in inflammatory processes which lead to the loss of the alveolar bone and the connective tissue in periodontal diseases.^17-19^ As a consequence of periodontitis, periodontal tissues are locally damaged and a large area of epithelium becomes wounded, allowing the exchange of products between the bacteria and the host.^20^

In a 30-year prospective cohort study involving 1112 participants, it was established that patients with worse plaque and periodontal indices at study onset were more likely to develop COPD.^24^ A study by Si et al^3^ identified the PI index as the sole factor dependent on periodontal health in COPD patients. Likewise, the current study confirmed that the PI index had a significant correlation with COPD variables, namely CAT, FEV1 and FVC. Since plaque accumulation is the primary precondition for periodontitis, the plaque index is obviously linked with the severity of COPD. Nevertheless, the exact mechanism to explain the relationship between oral hygiene/dental plaque and COPD remains largely unknown.^3^

Leuckfeldet al^18^ reported that an AL index >4mm is strongly correlated with COPD. In the present study, PD was the only index that exhibited a significant correlation with the FEV1/FVC ratio, which isa COPD variable. Generally, the aforementioned indices tend to increase as a result of periodontal diseases. Furthermore, periodontal diseases induce changes to the oral environment. The resultant conditions may facilitate the colonization and diffusion of respiratory pathogens on the oral mucosa.^21, 22^ Therefore, the mutual impact of periodontal variables on respiratory variables can be well justified.

Some studies have shown that COPD becomes more severe as a result of poor hygiene and quality of life.^23^ A study including a sample population of the Chinese revealed that individuals with inadequate knowledge of health, who are predisposed to unhealthy behavior and fail to observe oral hygiene, are at greater risk of developing COPD.^3^ In the present study, the CAT index showed a significant relationship with AL and PI variables.

A study by Peter et al^24^ showed that periodontal variable trends deteriorate with a decline in FEV1, indicating a negative correlation between periodontal variables and FEV1 values. However, this index showed a significant correlation with CAT, PD and GI variables but no significant correlation with PI and OHI indices.

These findings are not consistent with the results of the present study, according to which FEV1 showed a significant negative correlation with AL and PI while having no significant relationship with GI and PD variables. However, there were similarities in findings with the study conducted by Katancik et al^6^ in terms of some indices. They reported that FEV1 index was significantly correlated with GI and AL variables, but showed no significant correlation with PD. The existing conflicts and contradictions between these findings might be due to difference in the precision level of the spirometer devices used in various studies, varying degrees of accuracy in measurement of the periodontal variables, limited sample size or other confounding factors.

A study by Kucukcoskunet al^25^ showed that poor hygiene has a strong positive correlation with the severity of COPD, while being independent from age, gender and BMI. Similarly, the gender variable had no significant impact on the present study’s variables, except for GI. The higher GI index in women could be explained by hormonal changes and their effects on the gingiva.^26^

The strong link between the tobacco use and periodontal diseases, as well as COPD, has been verified by numerous studies. Thus, this particular element could serve as a confounding factor when the relationship between periodontitis and COPD is under investigation.^5^ In the current study, FVC was the only heightened variable among those who smoked >10 cigarettes a day compared to non-smokers.

The current study is the first comprehensive research to investigate the relationship between periodontal variables and respiratory indices with the aim to examine the link between the severity of COPD and periodontal diseases.

Considering the relationship between periodontal variables and respiratory indices in the course of COPD, early treatment of periodontal diseases, which can have a positive impact on lungs in terms of respiratory indices and volume, might considerably reduce the severity of COPD.

Moreover, paying more serious attention to periodontal hygiene, in conjunction with standard COPD treatment measures, can help control the severity of the condition. This can also prevent the incidence of relapse attacks, which commonly occur in the severe and very severe stages of COPD. It is recommended to perform further cohort studies and publish case reports in the future in order to confirm the findings of the present study, as well as to improve understanding of the cause-and-effect relationship between the variables mentioned in this study.

## Conclusion

Based on the results of this study, in view of the relationship between periodontal variables and respiratory indices in the course of COPD, early treatment of periodontal diseases might considerably reduce the severity of COPD.

## Competing interests

The authors declare no competing interests with regards to the authorship and/or publication of this article.

## Ethics approval

All procedures performed in this study involving human participants were in accordance with the ethical standards of the institutional research committee and with the 1964 Helsinki declaration and its later amendments or comparable ethical standards(Ethical registration number: IR.mums.sd.REC.1394.161.)
